# Molecular Research on Diabetes

**DOI:** 10.3390/ijms26051873

**Published:** 2025-02-21

**Authors:** Alessandra Puddu, Davide C. Maggi

**Affiliations:** Department of Internal Medicine and Medical Specialties, University of Genoa, 16132 Genoa, Italy; davide.maggi@unige.it

This Special Issue of the *International Journal of Molecular Sciences* collects the latest research on different biological processes and molecular mechanisms that cause diabetes. Diabetes is a metabolic disease characterized by hyperglycemia. The worldwide incidence of diabetes, particularly Type 2 Diabetes, accounting for 85–90% of all diabetes cases, has been steadily rising over the past few decades, becoming a major public health concern [1,2]. Given that diabetes raises the risk of microvascular and neurological disorders and contributes significantly to the pathophysiology of diabetes complications, this highlights the urgent need for global efforts in prevention, early detection, and management [3]. Diabetes can occur due to a variety of factors, with insulin resistance and β-cell dysfunction being the primary causative abnormalities. Moreover, several mutations in the genes important for glucose homeostasis and β-cell development have been linked to the progress of hyperglycemia. In addition, environmental factors may influence the incidence of hyperglycemia.

Recent diabetes research has allowed for advancements in insulin therapy, improving both insulin formulation, which means patients can respond more efficiently to changes in glucose levels, and ways of administration [4]. The development of wearable and implantable smart devices, such as glucose sensors and insulin pumps, has allowed for continuous glucose monitoring in conjunction with automated insulin delivery, helping to maintain glucose levels so that they are within target ranges [5]. These devices are often supported by software using Artificial Intelligence, thus helping to optimize insulin doses, based on continuous glucose monitoring and lifestyle factors, and to personalize therapy [6]. Moreover, the discovery of new classes of drugs, in particular incretin analogous and SLGT2 (Sodium-Glucose Transport Protein 2) inhibitors, has improved the management of glucose control in patients with Type 2 Diabetes, also contributing to reducing weight loss and the risk of cardiovascular disease [7]. Advances in genetic research have helped us to identify genes and other biomarkers involved in the pathogenesis of diabetes, which may improve the early detection of diabetes risk and help to refine diagnoses [8]. Several ongoing studies are focused on preserving pancreatic beta cell function and regenerating these cells in diabetic patients [9]. However, despite promising results in vitro, there are still many difficulties in translating them into clinical practice. In addition, despite the progress in the pharmacological treatment and management of diabetes, many questions remain unresolved. In particular, although several studies have aimed to elucidate the molecular mechanisms underlying the development of diabetes and its complications, their precise pathophysiology is not completely understood. This Special Issue showcases articles that try to address some of these gaps in our knowledge.

The contributions contained within this Special Issue evidence a great interest in exploring diabetes and related conditions, ranging from genetic findings to new therapeutic approaches. Genetic studies aim to characterize and identify different types of diabetes and to define the optimal therapeutic approach. In one study, a novel genetic variant in Hepatocyte nuclear factor-1-beta (HNF1B), a gene involved in transcriptional regulation, was linked to Maturity Onset Diabetes of the Young (MODY) in a family with renal cysts [10]. Moreover, Hasballa and Maggi highlighted that some genes involved in the differentiation and function of pancreatic beta cells have been identified as new potentially MODY-causal genes, even if they do not act monogenically and have no direct genotype–phenotype correlation [11]. A study on Latent Autoimmune Diabetes in Adults (LADA) in southeastern Mexico identified a significant genetic association with the single-nucleotide polymorphism (SNP) rs7305229, which affects the presence of glutamate decarboxylase autoantibodies [12]. In addition, exploring the SNPs associated with gestational diabetes (GDM) may also contribute to reducing the risk of future onset of diabetes in mothers and metabolic disorders in their children [13]. These findings may also be important for preventing deficiencies in memory and learning and in the development of pharmacological treatment, given that GDM caused hippocampal alteration in the offspring of diabetic rats [14]. Beyond genetic variants, this Special Issue also highlights the importance of exploring post-transcriptional events that may regulate the expression of important factors involved in the cytotoxicity of pancreatic beta cells [15]. On the other hand, improving islet isolation protocols, particularly in cases requiring the isolation of islets from live human donors, can provide valuable models for studying the physiology of islets and facilitate preclinical research into diabetes treatment [16]. Likewise, research based on human models of induced pluripotent stem cells (iPSCs) may make an important contribution to the field of regenerative medicine, also potentially providing a powerful tool for unraveling the activation of pathophysiologic pathways in models of human diseases [17,18]. For instance, Querio et al. demonstrated that iPSCs derived from cardiomyocytes respond to insulin by inducing the membrane translocation of Glucose transporter 4 (GLUT4), thus retaining the features of adult cardiomyocytes [19]. The availability of iPSCs that replicate the metabolic characteristics of adult cardiomyocyte represents a crucial aspect for investigating the various pathological and metabolic conditions associated with insulin resistance and diabetes. This is of particular importance given that cardiovascular diseases are the leading causes of morbidity and mortality in individuals with Type 2 Diabetes mellitus [20]. Interestingly, Batten et al. hypothesized that post-translational modifications, such as O-GlcNAcylation, may have a role in increasing thrombotic risk in individuals with poor glycemic control [21]. Additionally, the risk of cardiovascular disease in diabetic patients is associated with increased neutrophil counts and the resulting low-grade inflammation [22,23]. In support of this, levels of circulating low-density neutrophils (LDNs), which normally represent less than 2% of neutrophils in healthy individuals and are potent producers of cytokines and neutrophil extracellular traps, have been shown to be elevated in individuals with Type 2 Diabetes [24].

In conclusion, this Special Issue of the *International Journal of Molecular Sciences* focuses on significant advances in the understanding, treatment, and prevention of diabetes. It provides a comprehensive overview of the latest research on genetic findings, novel cellular models for studying mechanisms involved in the pathogenesis of diabetes in vitro, and strategies for developing therapeutic interventions to reduce the risk of cardiovascular diseases and the onset of diabetes during pregnancy, as well as metabolic and neuronal disorders in offspring. Additionally, new blood markers associated with diabetes have emerged. Future research on diabetes should focus on furthering our understanding of the disease and facilitating innovations to achieve more personalized approaches for the prevention and treatment of diabetes, thus improving quality of life for millions of patients.
